# Two predicted α-helices within the prion-like domain of TIAR-1 play a crucial role in its association with stress granules in *Caenorhabditis elegans*

**DOI:** 10.3389/fcell.2023.1265104

**Published:** 2023-12-15

**Authors:** D. A. Fuentes-Jiménez, L. S. Salinas, E. Morales-Oliva, V. A. Ramírez-Ramírez, M. Arciniega, R. E. Navarro

**Affiliations:** ^1^ Departamento de Biología Celular y Desarrollo, Instituto de Fisiología Celular, Universidad Nacional Autónoma de México, Mexico City, Mexico; ^2^ Departamento de Bioquímica y Biología Estructural, Instituto de Fisiología Celular, Universidad Nacional Autónoma de México, Mexico City, Mexico

**Keywords:** stress granules, TIA1, TIAR1, TIAR-1, stress, apoptosis, *C. elegans*

## Abstract

Stress granules (SGs) are sites for mRNA storage, protection, and translation repression. TIA1 and TIAR1 are two RNA-binding proteins that are key players in SGs formation in mammals. TIA1/TIAR have a prion-like domain (PrD) in their C-terminal that promotes liquid-phase separation. Lack of any TIA1/TIAR has severe consequences in mice. However, it is not clear whether the failure to form proper SGs is the cause of any of these problems. We disrupted two predicted α-helices within the prion-like domain of the *Caenohabditis elegans* TIA1/TIAR homolog, TIAR-1, to test whether its association with SGs is important for the nematode. We found that *tiar-1* PrD mutant animals continued to form TIAR-1 condensates under stress in the *C. elegans* gonad. Nonetheless, TIAR-1 condensates appeared fragile and disassembled quickly after stress. Apparently, the SGs continued to associate regularly as observed with CGH-1, an SG marker. Like *tiar-1*-knockout nematodes, *tiar-1* PrD mutant animals exhibited fertility problems and a shorter lifespan. Notwithstanding this, *tiar-1* PrD mutant nematodes were no sensitive to stress. Our data demonstrate that the predicted prion-like domain of TIAR-1 is important for its association with stress granules. Moreover, this domain may also play a significant role in various TIAR-1 functions unrelated to stress, such as fertility, embryogenesis and lifespan.

## Introduction

The highly conserved RNA binding proteins TIA1 (T-cell-restricted intracellular antigen 1) and TIAR (TIA1-related protein) have been thoroughly studied in mammals because they play a key role in the assembly of the biomolecular condensates better known as stress granules (SGs) (Kedersha et al., 1999; Kedersha et al., 2000). Stress granules are transitory cytoplasmic ribonucleoprotein condensates that are formed in eukaryotes under stressful conditions such as nutrient starvation, heat shock, high osmolarity, oxidative stress and/or DNA damage (Riggs et al., 2020). In addition to their role in SGs formation, TIA1 and TIAR participate in splicing, translational repression, and the relocalization of mRNAs. At their N-terminus, these proteins have three RNA-Recognition Motifs (RRM1-3) that allow them to interact with specific mRNA sequences localized at splice sites or the 3′ UnTranslated Region (UTR) (Förch et al., 2000; Dixon et al., 2003; López de Silanes et al., 2005; Izquierdo et al., 2011). At their C-terminus, TIA1 and TIAR possess a low complexity domain (LCD) enriched in residues commonly found in prions and prion-like domains (PrD) that play key roles in SGs nucleation (Rayman and Kandel, 2017).

The sole expression of TIA1 PrD is sufficient to trigger the formation of condensates, although these condensates are smaller than the well-characterized SGs (Gilks et al., 2004). In contrast, the overexpression of TIA1 PrD causes a dominant-negative effect that blocks SGs formation (Gilks et al., 2004). Expression of the human full-length TIA1-EGFP, and a truncated version that only expresses the PrD (290–386) of TIA1-EGF, is sufficient to form condensates *in vivo* (in mouse neuroblastoma cells [N2a]) and *in vitro* (after 10% w/v PEG addition and salt or temperature changes). These condensates show a fast Fluorescence Recovery After Photobleaching (FRAP), revealing that human TIA1 can undergo liquid-liquid phase separation (Ding et al., 2021).

Mutations in prolines residues within the TIA1 PrD are observed in the TIA1 proteins of patients with Amyotrophic Lateral Sclerosis (ALS) and Fronto-Temporal Dementia (FTD). *In vitro*, PrD TIA1-EGF-expressing proteins that carry mutation on some of these proline residues can form droplets that, depending on the type of mutation, can be either big, small, and/or have different shapes. These droplets are in general less prompt in recovering after photobleaching, suggesting that prolines are important in supporting the formation of stress granules, in rendering them more fluid or liquid (Ding et al., 2021). Additionally, condensates formed by the TIA1 protein with proline mutation do not clear as fast as wild-type TIA1 granules after stress.

In *Caenorhabditis elegans*, there are three TIA-1/TIAR homologs. TIAR-1 is expressed in the whole organism, associates with SGs and plays an important role in their assembly both in the soma and the germline (Rousakis et al., 2014; Huelgas-Morales et al., 2016). Animals lacking TIAR-1 exhibit defects in fertility, embryogenesis, larval development, lifespan, germ cells apoptosis, germline stress resistance, and are sensitive to oxidative stress and Ultraviolet (UV) radiation (Silva-García and Estela Navarro, 2013; Rousakis et al., 2014; Huelgas-Morales et al., 2016; Andrusiak et al., 2019). TIAR-2 localizes to stress granules in soma and germline, but is mainly required for stress-granules assembly in the soma and aggregates in senescent pharyngeal muscles (Gallo et al., 2008; Rousakis et al., 2014; Huelgas-Morales et al., 2016; Andrusiak et al., 2019). TIAR-2 is involved in the inhibition of axon regeneration mediated by granule formation (Lechler and David, 2017; Andrusiak et al., 2019). TIAR-3 is not required for SGs assembly, probably because it lacks a PrD; however, it has not yet been as extensively studied as its homologs (Rousakis et al., 2014; Huelgas-Morales et al., 2016).

To date, it is not well understood whether the role of TIAR-1 in SGs condensation has a correlation with other aspects of its function. We hypothesized that TIAR-1’s PrD is necessary for its condensation; therefore we mutated this domain to study the contribution of TIAR-1 to condensation and its other roles in the biology of the organism. To disrupt the prion-like domain of TIAR-1, we selected a hypothetical α-helix of 29 residues within a predicted aggregation-prone region in the TIAR-1 carboxy-terminus. We changed six interspersed residues within this hypothetical α-helix into proline to reduce its structure stability. We will refer to this mutant as the *tiar-1* PrD6 mutant. As expected, the mutant protein condensed less efficiently in SGs of tiar-1 PrD6 animals than its wild-type transgene counterpart in response to stress. This result demonstrates that this domain plays an important role in TIAR-1 condensation. Additionally, and similiar to the *tiar-1* null allele (Huelgas-Morales et al., 2016), the *tiar-1* PrD6 mutant protein’s association to SGs in oocytes was not affected, supporting the idea that these granules are assembled by a different mechanism. We observed that TIAR-1 condensates that form in the PrD-mutant animals disassembled faster after stress than those formed in wild-type. Additionally, condensates in *tiar-1* PrD mutant animals rapidly dissolved in the presence of 1-6 hexanediol, suggesting that their interactions are weaker than those formed by the wild-type protein. Unexpectedly, the SGs marker CGH-1 continues to associate with SGs in *tiar-1* PrD mutant animals, suggesting that only TIAR-1 condensation is affected when its PrD is mutated.

The *tiar-1* PrD6 mutant animals, like null *tiar-1* animals, showed defects in fertility and a shorter lifespan. Unexpectedly, PrD6-mutant animals demonstrated higher embryonic lethality than null *tiar-1* animals. In contrast to the null *tiar-1* animals, PrD6-mutant animals were no longer sensitive to oxidative stress and UV radiation and exhibited normal response to stress-induced apoptosis. Our results demonstrate that the PrD domain of TIAR-1 might be necessary for the fertility, embryonic development, and lifespan, but not for other functions related to germ-cell apoptosis and stress resistance.

## Materials and methods

### Strains

**FIGURE 1:**
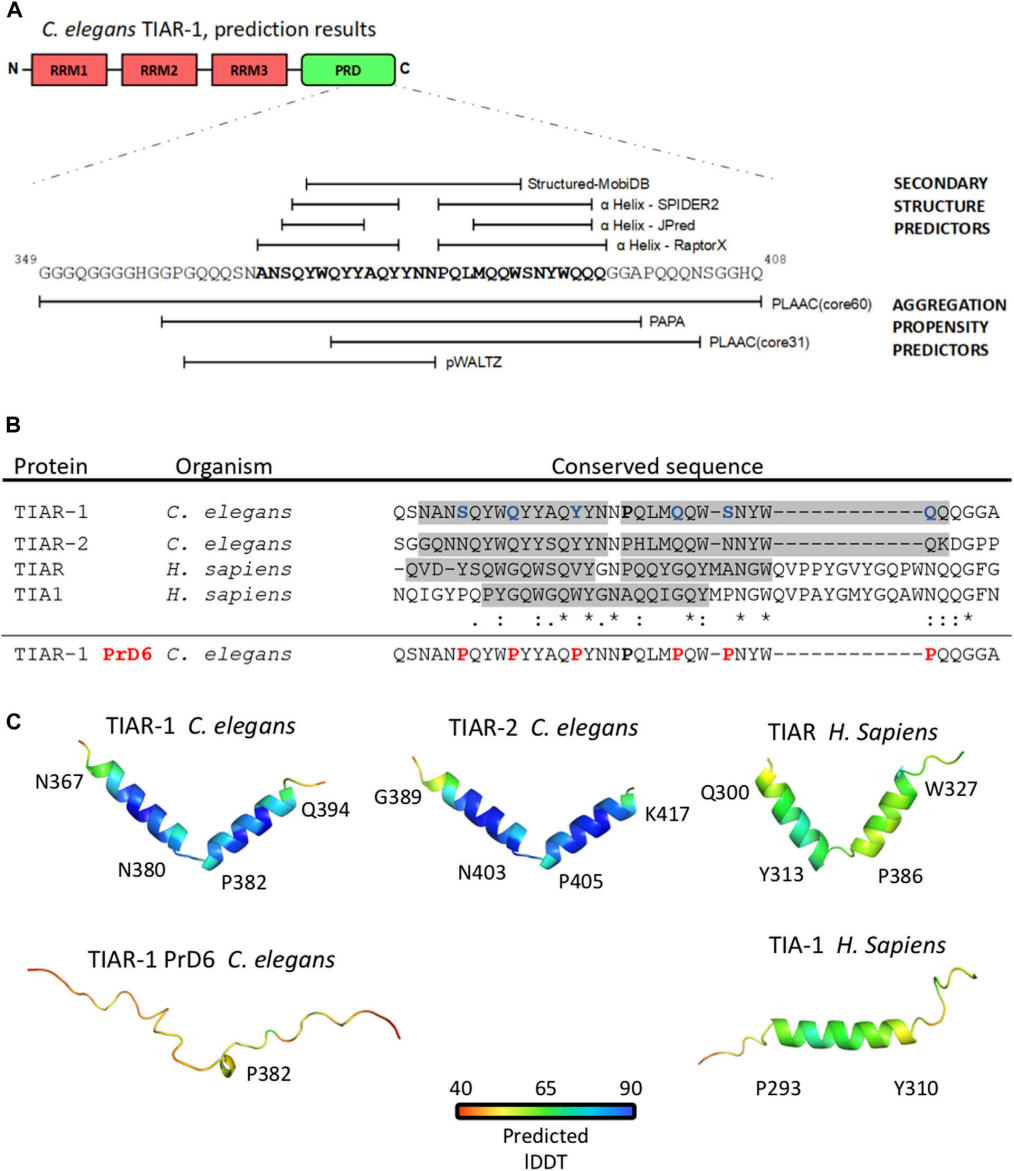
The substitution of 6 residues in the prion-like domain fo TIAR-1 with proline disrupted a double *α* helix structure. **(A)** Representation of a segment of TIAR-1 prion-like domain with predictions of its structure (upper) and aggregation propensity (lower) based on its sequence. The webservers employed for this purpose are described in the figure. **(B)** Multiple sequence alignment (MSA) with Clustal O of the conserved sequences found with COBALT. Shaded regions highlight the sequence segments forming the *α* helices observed in the molecular models represented in **(C)**. In the wild-type version of *C. elegans* TIAR-1::GFP fusion protein, the residues S369, Q373, Q377, Q386, S389, and Q393 were mutated to prolines (shown in red). The mutated sequence was not included in the analysis. Asterisk (*) indicates a single fully conserved residue, colon (:) conservation between groups of strongly similar properties, period (.) indicates conservation between groups of weakly similar properties. **(C)** Molecular models of the TIAR-1 prion-like domain of a set of TIAR-1 homologous proteins shown in ribbon representations. Models are colored according to the plDDT (Predicted Local Distance Difference Test). For all models, the average plDDT is above 50%. For each model, the aminoacid labels indicate the *α* helix starting and ending position.

*C. elegans* strains were maintained at 20°C on NGM-Lite and fed with the *Escherichia coli* strain OP50-1 for the majority of the experiments, except for when RNAi were used, the strain employed was HT115 (DE3) (Brenner, 1974; Timmons et al., 2001). The following strains were employed: wild-type Bristol N2, DG3922–*tiar-1*(*tn1545[tiar-1::gfp::tev::s]*), DG3929–*tiar-1(tn1543)* available at the *Caenorhabditis* Genetics Center (CGC, University of Minnesota, United States). Strain PHX4286, generated by SunyBiotech (Fujian, China) upon our request, was derived from the parental strain DG3929. The modification of the PrD domain, as depicted in Figure 1, was achieved using CRISPR-Cas9, resulting in the genotype *tiar-1*(*syb4286*[*tn1545*(S369P, Q373P, Q377P, Q386P, S389P and Q393P)]). Upon arrival, strain PHX4286 was outcrossed three times and the modified region was amplified by PCR and corroborated by DNA sequencing.

### Brood size and embryonic lethality assays

Mid-L4 larval stage hermaphrodites of each strain were individually placed onto NGM-lite plates seeded with OP50-1. The animals were transferred daily to fresh NGM-lite plates seeded with OP50-1 until they ceased laying embryos (approximately 3 days). Plates were scored for dead embryos and surviving progeny after 24 h of moving the parental hermaphrodite. Embryos not hatching within 24 h after being laid were considered dead. The total number of progeny per animal (brood size) of each strain was counted and plotted.

### Stress-granule inducing conditions

Synchronized L1 animals were grown at 20°C on NGM-lite plates seeded with OP50-1 bacteria and were grown until they were one-day-old adults. The population was separated into stressed and control groups. For heat shock, one-day-old hermaphrodites were transferred onto seeded plates, which were then sealed with Parafilm, and placed in a controlled temperature water bath at 31°C for 3 or 4 h. The control (no stress) group plates were kept in the incubator at 20°C. For heat-shock recovery experiments, the plates containing heat-shocked animals were returned to a temperature of 20°C and were thus maintained for 6 h after stress. For the starvation experiments, the animals were transferred to glass Petri dishes containing liquid M9 supplemented with cholesterol (5 μg/mL) and no bacteria, and kept at 20°C for 4–6 h. For control, animals were transferred to glass Petri dishes containing liquid M9 supplemented with cholesterol (5 μg/mL) and freshly grown OP50-1 (at a 1/20 dilution of a culture with 0.66 absorbance at 600 nm).

To quantify stress granules in the gonad after each treatment, 10–20 animals were placed on 2% agar pads prepared in M9 containing a drop of M9 and 0.01% tetramisole. Once the animals stopped moving, a coverslip was placed on top of the sample and was observed under the microscope. Image acquisition was performed using a Nikon Eclipse E600 microscope equipped with an AxioCam MRc 5 camera and Zeiss AxioVision software or a confocal Zeiss LSM8001 microscope with a ×40 objective lens (NA = 1.4), at 1,024 × 1,024 pixels and the Zeiss AxioVision software. When needed, picture brightness and contrast was adjusted to reduce background using the FIJI image processing package (Schindelin et al., 2012). At least two independent experiments were conducted with several animals observed for each condition and time point.

### Disassembly of SGs in the presence of 1,6-hexanediol

Animals exposed to heat shock (4 h at 31°C), as described previously, were mounted on one drop of egg buffer (control) or in the presence of 2, 3 or 5% of 1,6-hexanediol (SIGMA-ALDRICH Cat. Number 240117) diluted in egg buffer that was placed on the top of a slide pretreated with poly-L-lysine (SIGMA-ALDRICH Cat. P8920). The animal´s gonads were dissected and covered with a square coverslip and observed immediately after extrusion under the fluorescence microscope, in order to score this for presence of SGs. At least two independent experiments were conducted with several animals observed under each condition. The images of living animals were acquired on a Nikon Eclipse E600 microscope equipped with an ZEISS AxioCam MRc 5 camera and Zeiss Axio Vision software or a confocal Zeiss LSM8001 microscope with a ×40 objective lens (NA = 1.4), at 1,024 × 1,024 pixels, and the ZEISS AxioVision software.

### Immunostaining

To visualize germline SGs, immunostaining against CGH-1 and GFP was performed as previously reported by (Huelgas-Morales et al., 2016) with some modifications. The gonads of one-day-old animals were dissected, freeze cracked, fixed in cold methanol for 1 min, and then treated with 3.3% paraformaldehyde for 15 min. The samples were washed twice with PBT, and were then blocked in PBT containing 30% of normal goat serum (NGS; Sigma-Aldrich, St. Louis, MO) for 30 min. Primary antibody incubation was performed overnight at 4°C with rabbit anti-CGH-1 (1:1000) (Boag et al., 2005) and mouse anti-GFP (1:50) (A11120 from Molecular Probes, Eugene, OR). Secondary antibody incubations were performed for 2 h at room temperature with Alexa Fluor 594-conjugated anti-rabbit IgG and Alexa Fluor 488-conjugated anti-mouse IgG (1:100; H + L; A11001, Molecular Probes, Eugene, OR). To detect DNA, 1 ng/ul 4′,6-diamidino-2-phenylindole dihydrochloride (DAPI) was used. Vectashield (Vector laboratories, Burlingame, CA) was added to avoid photo bleaching before sealing the sample.

### Germ cell apoptosis assays

We used RNA interference (RNAi) in the *ced-1* gene to facilitate visualization of cell corpses (Salinas et al., 2006). Compared to wild-type, *ced-1*(RNAi) animals (as well as *ced-1*-mutant animals) retained apoptotic cells for longer periods of time due to defects in their corpse degradation machinery. We induced *ced-1* double-stranded RNA in liquid cultures using the feeding bacteria strain HT115 (DE3) following standard procedures, as we previously reported in (Salinas et al., 2006). Control bacteria carrying an Empty Plasmid (EP) or the *ced-1* clone were cultured under the same conditions. After the induction of dsRNA, 1-mL aliquots of bacteria carrying an EP or *ced-1* dsRNA were frozen in liquid nitrogen and maintained at −70°C. When needed, aliquots were thawed, centrifuged, and the pellet was resuspended in 100 µL of LB containing antibiotics and IPTG. Fifty µl of bacteria were seeded onto each 60-mm plate of NGM containing antibiotics. The plates were used the same day as soon as the bacteria dried. A total of 100–120 synchronized L1 larvae were set in place for each 60 mm plate containing dsRNA and kept at 20°C. L4 larvae were moved onto a fresh RNAi plate and incubated for another 24 h. One-day-old control (EP) or *ced-1(RNAi)* animals of each strain were maintained under normal growth conditions at 20°C (control) or exposed to 3 h at 31°C (Salinas et al., 2006). For starvation experiments, one-day-old animals were transferred into M9 containing cholesterol (5 μg/mL) and freshly grown OP50-1 (a 1/20 dilution of a culture with 0.66 absorbance at 600 nm) at 20°C (control) or into M9 containing cholesterol (5 μg/mL) without bacteria for 4 h (Salinas et al., 2006). For senescence apoptosis, animals were maintained under normal growth conditions at 20°C. Each day, for the following 4 days, 10–20 animals were mounted on agar pads that contained one drop of 40 mM NaN3 (for immobilization) diluted in M9. The animals were observed using Nomarski optics to visualize cell corpses on a Nikon Eclipse E600 microscope equipped with an ZEISS AxioCam MRc 5 camera and Zeiss Axio Vision software. Excel (Microsoft) was used to calculate average standard error of the mean (S.E.M.) and *p*-values (by means of the Student’s *t*-test).

### Stress resistance assays

To test the animals’ resistance to stress, we followed the conditions published by Rousakis et al. (Rousakis et al., 2014). Briefly, for UV resistance assays, 5-day-old adults were placed on 60 mm NGM-lite plates without bacteria, and irradiated at 0.2 J/cm^2^ on a UV Stratalinker (Stratagene, Champaign, IL, United States). After irradiation, animals were transferred onto NGM-lite plates seeded with OP50-1 and maintained at 20°C. After 72 h, the animals’ survival was determined. For oxidative stress, one-day-old adults were placed onto NGM-lite plates seeded with UV killed bacteria (OP50-1 irradiated at 0.99 J/cm^2^) and 5 mM sodium arsenite; plates were incubated at 20°C and the percentage of surviving animals was determined after 48 h. For osmotic stress, one-day-old adults were placed onto NGM-lite plates to which had been added with 400 mM NaCl, seeded with OP50-1. The animals were incubated at 20°C, and the percentage of surviving animals were maintained after 24 h. For each experiment, the number of surviving animals was determined by mobility or response-to-touch stimuli. Per assay, ≥100 hermaphrodite adults were evaluated for each strain.

### Lifespan assays

For these assays, hermaphrodites at the mid-L4 larval stage of each strain were placed onto NGM-lite plates seeded with OP50-1 bacteria and were maintained at 20°C throughout the course of the experiment. For the first 4 days, the animals were transferred onto new plates every 24 h to separate them from their progeny. The viability of the animals was scored daily until no animals remained. Animals that did not respond to stimulation by touch or loss of pharyngeal pumping were referred to as dead. Bagged, ruptured, or animals that had crawled off the plates were eliminated from the analysis. The total number of live vs. dead animals for each strain was quantified until the last dead animal was observed. At least two independent experiments were conducted with 700 animals in total for each indicated genotype. Lifespan and statistical analysis were performed using GraphPad Prism version 8.0.1 statistical software for Windows (www.graphpad.com).

### Bioinformatics

LCD sequences of *C. elegans* TIAR-1 and TIAR-2, and *Homo sapiens* TIA-1 and TIAR were obtained from their full protein Uniprot entries (https://www.uniprot.org/; Q95QV8, Q9U2F5, P31483, and Q01085), selecting the C-terminal region after the third RRM. We employed the structure predictor webservers of MobiDB 2.0 (Potenza et al., 2015), JPred4 (Drozdetskiy et al., 2015), Spider2 (Heffernan et al., 2016) and RaptorX (Wang et al., 2016) and the aggregation-prone predictor webservers of PAPA (Toombs et al., 2012), pWaltz (Sabate et al., 2015) and PLAAC (Lancaster et al., 2014). For these webservers, we conducted a benchmark test, with six random sequences of structured protein regions obtained from the RCSB-Protein Data Bank (https://www.rcsb.org/) and with the LCD sequences of *Homo sapiens* FUS, hnRNPA1, hnRNPA2B1, TDP43, *C. elegans* LAF-1 and *Saccharomyces cerevisiae* Lsm4p (not shown). For multiple alignments between the TIAR-1 predicted α-helix and other TIA1-family LCD sequences, we employed COBALT (Papadopoulos and Agarwala, 2007).

### Statistical analysis

Analysis was performed on GraphPad Prism version 8.0.1 for Windows statistical software (www.graphpad.com), assuming consistent Standard Deviations (SD). Defined gonad core and oocyte stress-granule phenotype comparisons between the two groups were tested by multiple Student’s *t*-test. Brood size and embryonic lethality were tested by one-way ANOVA and the Tukey multiple comparison test. Cell corpses per gonad, and survivability to stress were tested by two-way ANOVA and the Tukey multiple comparison test. Defined gonad core and oocyte stress-granule phenotype comparisons among more than two groups were tested by two-way ANOVA and the Dunnett multiple comparison test, assuming the “EV” condition as control for only-RNAi-treated conditions and the “no vector” condition for mutant and RNAi-treated mutant conditions.

### Data availability

Strains and materials will be provided upon request.

## Results

The disruption of the prion-like domain resulted in condensation and stability alterations of TIAR-1, compromising its association with SGs.

TIAR-1 possesses three RNA Recognition Motifs (RRM) in its N-terminus and a Prion-Like Domain (PrD) in its C-terminus, which are characteristic of TIA1/TIAR-related proteins (Figure 1A) (Silva-García and Estela Navarro, 2013; Waris et al., 2014). We decided to analyze the PrD of TIAR-1 (residues 313–408) to select specific sites to modify and potentially disrupt the capacity of TIAR-1 to condensate into SGs in the germline, as we previously reported (Huelgas-Morales et al., 2016). We considered a model suggested in the work of Sabate et al. (2015) in which the authors proposed that specific short amyloid-prone sequences in a protein may determine its ability to form prion-like condensates. We employed secondary structure and aggregation-prone predictors to analyze this region (see Materials and Methods). A region of 29 residues (367–395) of the TIAR-1’s PrD was predicted to be composed of two α helices (Figure 1A). The region covering the hypothetic structures also was considered prone to aggregation (Figure 1A).

We employed a multiple alignment tool, Clustal O ver. 1.2.4 (Madeira et al., 2022) to compare the hypothetical *α* helices on TIAR-1 (plus three residues located before and after the region selected, respectively) with the sequences of the PrD of TIAR-2 (a paralog on *C. elegans*) and TIA1 and TIAR (human homologs). Interestingly, this specific region is conserved between TIAR-1 homologs (Figure 1B). We observed that on TIAR-1’s predicted α helices, there is a region enriched in residues that are abundant in prion-like proteins (Q, N, Y, W, and S), which are the focus of the aggregation-prone predictors that fall into the *α* helices (Figure 1B). In particular, prion-related residues revealed a strong degree of identity across homologs.

Motivated by these findings, molecular models of these regions were built for 4 TIAR-1 homologs using AlphaFold2 (Jumper et al., 2021). Remarrkably, while TIAR-1 and its homolog protein TIAR-2 formed two α helices, similar to their human homolog TIAR, the human TIA1 only folded into one *α* helix (Figure 1C). The presence of this α helix composition is a rather uncommon feature observed in prion-like domains (Conicella et al., 2016; Conicella et al., 2020; Chiu et al., 2022).

To disrupt TIAR-1 hypothetical α helices in the PrD, we modified spaced residues into prolines, as this amino acid is known to inhibit secondary structures. Proline 382 was employed as a point of reference for modifying six residues at a distance of 3–5 amino acids between each of them (Figure 1B). A molecular model of the modified region predicted that the structure of this region was completely disrupted after the a substitutions into proline (Figure 1C). Altogether, these results suggest the following: 1) the alpha helix is an important element conferring prion-like properties, and 2) structural disruption, achieved through the incorporation of prolines, will result in a change in phenotype.

### The prion-like domain in TIAR-1 is important for both the condensation and stability of TIAR-1

We requested from the SunyBiotech Company (Fujian, China) a mutant animal with the indicated modifications on the strain DG3922, which carries a transgenic endogenous *tiar-1*(*tn1545[tiar-1::s::tev::gfp]*) conducted in (Huelgas-Morales et al., 2006). The Company denominated *tiar-1* mutated animals’ allele *syb4286* and the strain name is PHX4286 (see material and methods). We outcrossed the PHX4286 strain three times with N2 and later confirmed the substitutions by means of sequencing. We will refer to the Prion Domain 6 added prolines strain as the PrD6 mutant.

**FIGURE 2:**
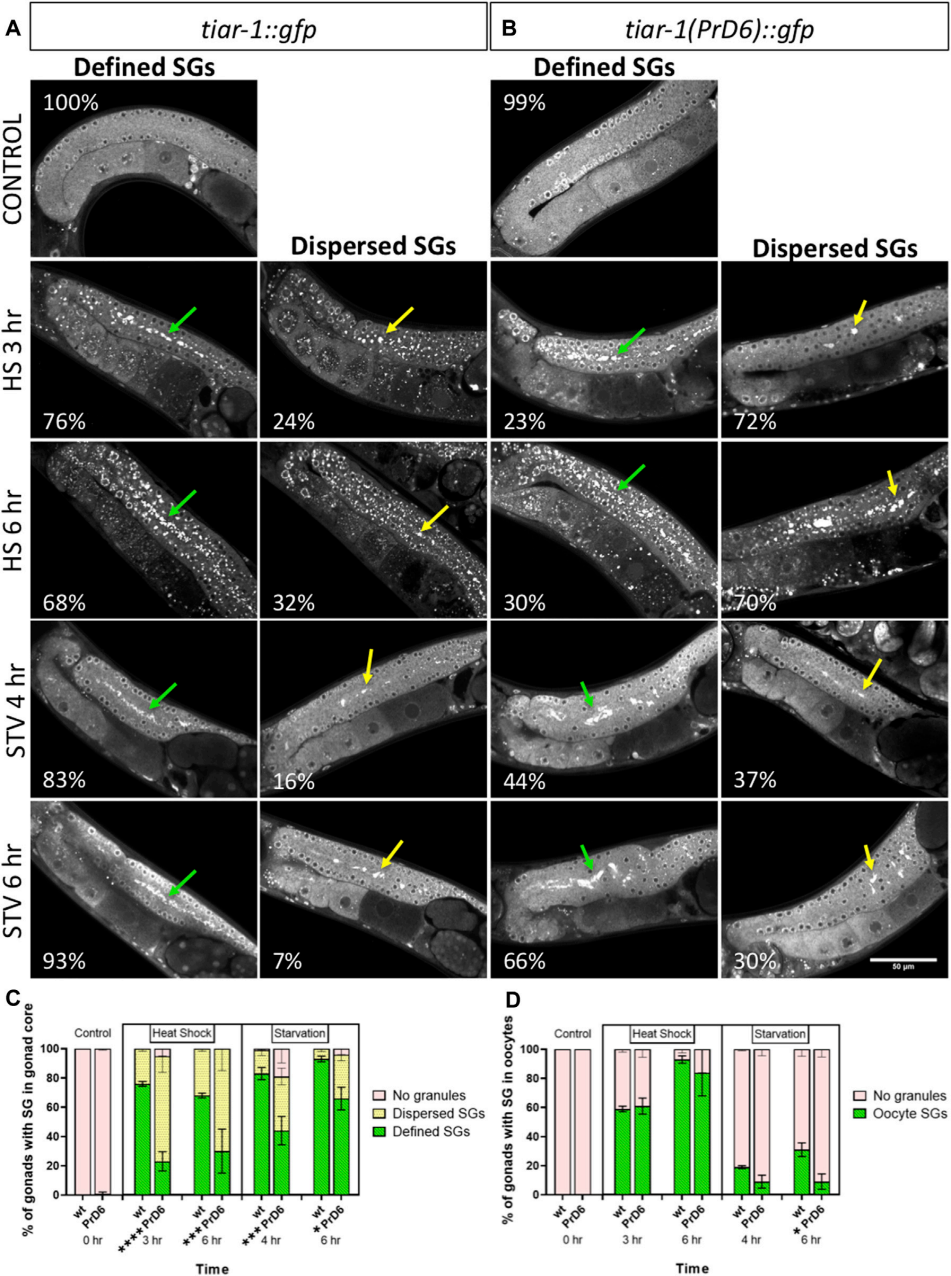
PrD6-mutant animals showed reduced TIAR-1′ condensation. One-day-old *tiar-1::gfp* (wt) and *tiar-1(PrD6)::gfp* hermaphrodites were exposed to control or stress conditions as indicated in materials and methods, anesthetized and observed under confocal microscopy for pictures of epifluorescence for SGs quantification. **(A, B)** pictures of gonads of each treatment. Gonads with no SGs are shown in control conditions, defined SGs are shown with green arrows and dispersed SGs are shown with yellow arrows. Inside each image, the percentage of gonads with each phenotype is indicated. Scale bar of 50 μm. **(C, D)** Graph showing the quantification of SGs for each condition in the gonad core **(C)** and oocytes **(D)** at indicated conditions. Four independent experiments were conducted with an *N* = 25 for each condition. Multiple *t*-test, assuming same scatter, were employed and the results are indicated as follow: ****p < 0.0001, ***p < 0.001, **p < 0.01, *p < 0.05.

Previously, we reported that *tiar-1* knockout (KO) animals did not form SGs in the gonad core when we used CGH-1 as a SGs marker (Huelgas-Morales et al., 2016). To test the TIAR-1 condensation efficiency of the PrD6 mutant animals, we exposed wild-type *tiar-1::gfp* (*tn1545*) and PrD6-mutant (*syb4286*) one-day-old hermaphrodites to 3 h of heat shock (at 31°C) or 4 h of liquid starvation. The overall expression and distribution of reporters in wild-type *tiar-1::gfp (tn1545)* and the PrD6 (*syb4286*) mutant animals were similar in control conditions (Figures 2A, B). During heat shock, the majority of the *tiar-1::gfp* wild-type nematodes (76%) showed that TIAR-1 condensates aligned along the center of the distal gonad core, mostly contacting each other and forming a line (green arrows). For the purposes of our analysis, we considered these condensates as defined SGs (Figures 2A, C). We also observed certain less organized and dispersed SGs on the gonad core of some animals (24%) that we classified as dispersed SGs (yellow arrows) (Figures 2A, C). In the PrD6-mutant animals, we observed a decrease in the proportion of gonads with defined SGs (23%); instead, more gonads showed dispersed SGs (72%) or no SGs (5%) (Figures 2B, C). To test whether there is a delay in SGs assembly in PrD6-mutant animals, we exposed the animals to a temperature of 31°C for 6 h. We observed a slight increase in defined SGs (30%) in PrD6 animals under this condition; however, the proportion of animals with dispersed SGs did not change (Figures 2B, C). Overall, we did not observe changes in the size or number of the SGs between the wild-type and PrD6-mutant animals.

We tested the requirement of TIAR-1′s PrD on gonad SGs formation during starvation (no bacteria for 4 or 6 h). The majority of the wild-type animals (*tiar-1::gfp*) formed well-defined SGs in the gonad core after 4 h of starvation (83%) (green arrows). Indeed, SGs formation in the wild-type was more efficient after 6 h of fasting (93%) (Figures 2A, C). In contrast, less than one-half of the PrD6 mutant animals exhibited well-defined SGs formation after 4 h of fasting (44%) (green arrows) (Figures 2B, C). The efficiency of SGs formation improved after 6 h (66%) in the PrD6 animals, although it did not reach the efficiency of the wild-type animals (93%) (Figures 2A–C). We did not observe significant changes in SGs formation in oocytes under heat shock or starvation in the PrD6-mutant animals (Figures 2A, B, D). Our results suggest that the prion-like domain is important for TIAR-1 condensation mainly during heat shock. It is possible that other factors might aid in condensing or relocating TIAR-1(PrD6)::GFP to the gonad core, especially in the case of extended starvation.

**FIGURE 3:**
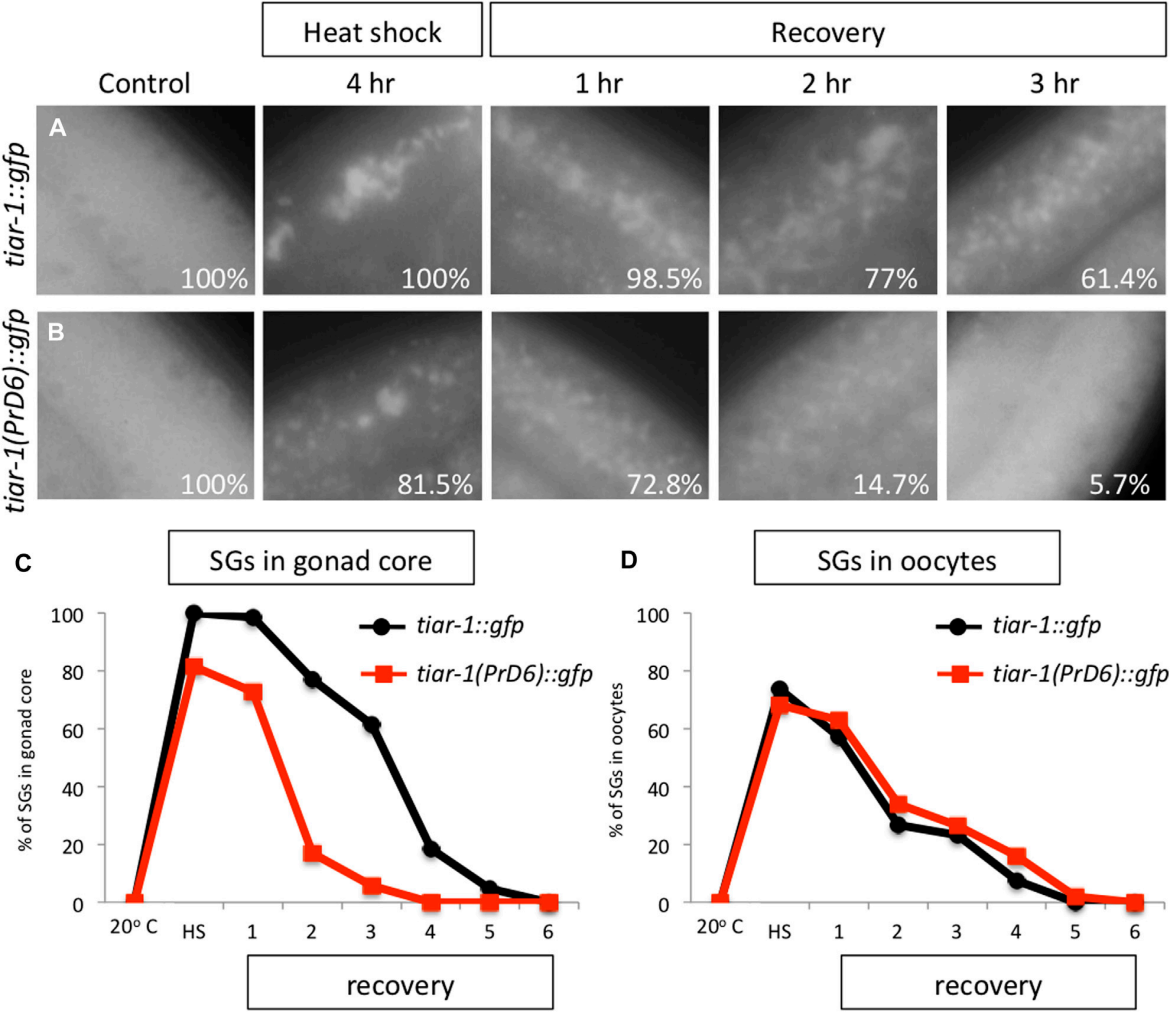
The TIAR-1 PrD6 mutated protein dissociated more rapidly from condensates during the recovery phase. **(A, B)** Pictures of selected areas of the gonad core of one-day-old *tiar-1::gfp* and *PrD6-*mutant animals exposed to heat shock (4 h at 31°C) and mounted under the epifluorescence microscope. The percentage of animals showing condensates is shown in each picture. After heat shock, animals were placed at 20°C for recovery, and mounted every hour to quantify condensates until these were no longer observed. **(C, D)** Graphs showing the percentage of animals showing condensates under the previously described conditions in the goand core **(C)** or in oocytes **(D)**. At least two independent experiments were conducted with an N of > 40 animals for each condition and time point. The average percentage of animals with visible granules is depicted in the graphs.

SGs are transitory and they disassemble when stress ends (Huelgas-Morales et al., 2016). To test the disassembling efficiency of the TIAR-1’s SGs formed in the PrD6-mutant animals, we subjected *tiar-1::gfp* and PrD6-mutant animals to 4 h of heat shock at 31°C for better SGs assembly. After stress, the animals were set in place at 20°C and mounted every hour to quantify the presence of TIAR-1 condensates in the gonad core and oocytes. For the purpose of these experiments, we quantified gonads with defined and dispersed granules as the same category. As expected, we did not observe TIAR-1 condensate formation under control conditions (Figures 3A–C). A total of 100% of *tiar-1::gfp* animals formed condensates in the gonad core after 4 h of heat shock (Figures 3A, C). After 5 h of recovery at 20°C, we nearly did not observe *tiar-1::gfp* animals with condensates in their gonads (Figure 3C). The majority of PrD6-mutant animals exhibited TIAR-1 condensate assembly (of both dispersed or defined SGs) after 4 h of heat shock (81.5%) (Figures 3B, C). However, these condensates disassembled rapidly, compared with those in wild-type animals, and in just 2 h, only 15% of animals showed TIAR-1 condensates (Figures 3B, C). We did not observe differences in oocytes condensates disassembly between *tiar-1::gfp* and PrD6-mutant animals; in both strains oocytes condensates disassembled with the same efficiency after 4 and 5 h of heat shock (Figure 3D). These data suggest that the condensates of TIAR-1 formed in animals with the PrD mutation have weaker interactions, and that is why the TIAR-1 PrD6 fusion protein returned to the cytoplasm faster than the wild-type.

**FIGURE 4:**
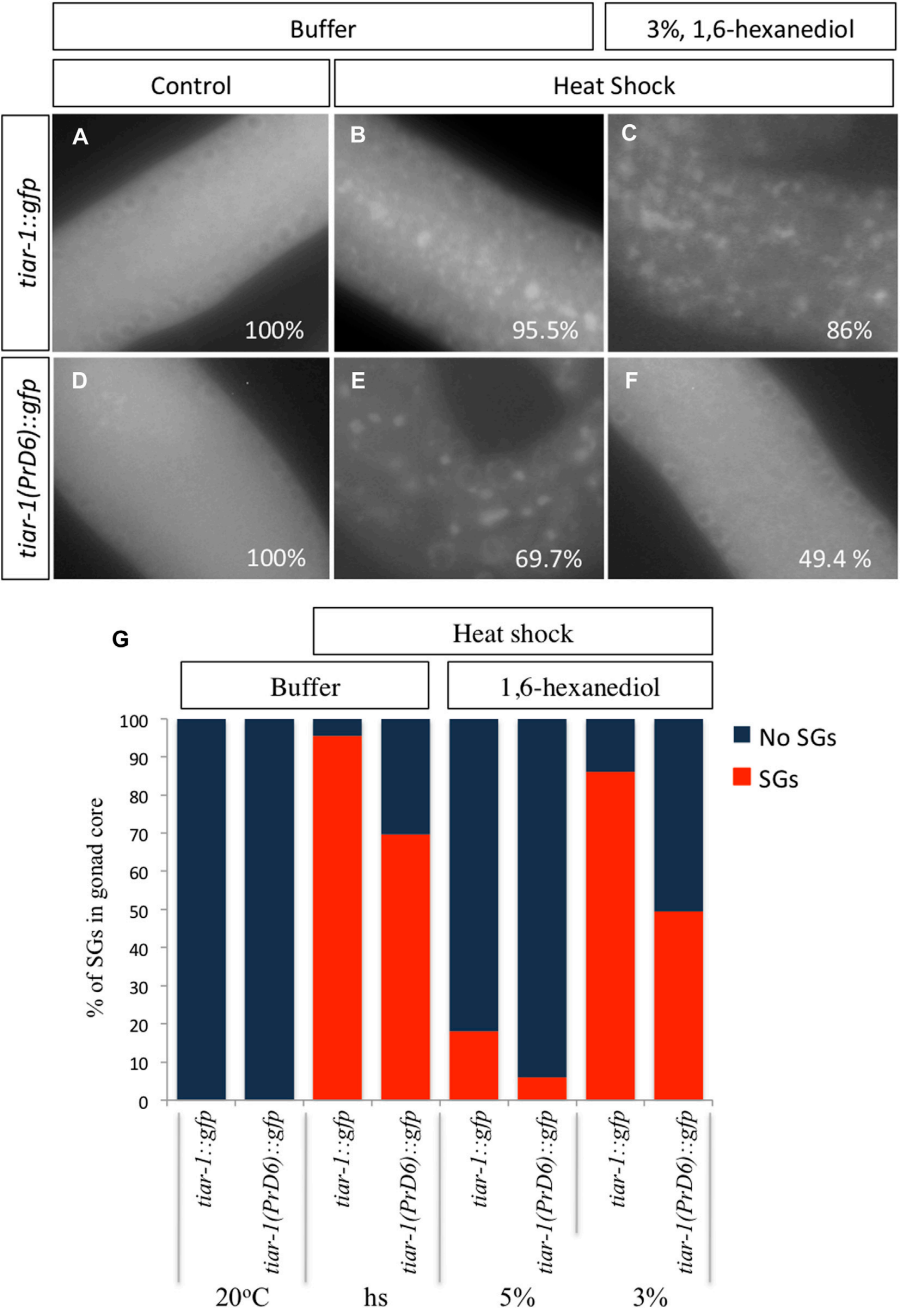
The condensates formed by the TIAR-1 PrD6 mutated protein exhibited weaker interactions compared to the wild-type protein. **(A–F)** Pictures of selected areas of the gonad core were taken. One-day-old *tiar-1::gfp* and *PrD6-*mutant hermaphrodites that were exposed to heat shock (4 h at 31°C) or kept at 20°C (control). After treatment, gonads were extruded on buffer **(A, B)** or in presence of 3% 1,6-hexanediol. Samples were covered with a coverslip and observed immediately under the epifluorescence microscope to score for condensates. The percentage of animals showing condensates is shown in each picture. **(G)** Graph showing the percentage of animals that showed condensates (SGs) in the gonad core for each condition. At least two independent experiments were performed, each involving minimum of 50 animals for each condition.

1, 6-Hexanediol disrupts the weak interaction associated with liquid biomolecular condensates (Kroschwald and Alberti, 2017). To test the strength of the condensates of the PrD6-mutant TIAR-1, we exposed *tiar-1::gfp* and PrD6-mutant animals to heat shock (4 h at 31°C). After stress, the animals’ gonads were dissected in egg buffer with or without 1, 6-hexanediol, and the samples were observed under the epifluorescence microscope immediately thereafter. For the purpose of these experiments, we quantified TIAR-1 condensates as defined or dispersed granules in the same category. We did not observe TIAR-1 condensates under control conditions (20°C, no 1, 6-hexanediol) in any of the tested strains (Figures 4A, D, G and Supplementary Figure S1A). After heat shock, the majority of the wild-type animals formed TIAR-1 condensates (95.5%), while 69.7% of PrD6 animals showed them (Figures 4B, E, G and Supplementary Figure S1B). The majority of the condensates in the wild-type *tiar-1::gfp* animals dissolved immediately in the presence of 5% of 1, 6-hexanediol (82%) (Supplementary Figure S1C), however, most of the condensates were still observed in 3% of this alcohol (86%) (Figures 4C, G). In PrD6-mutant animals, 49.4% of the gonads maintained condensates in the presence of 3% of 1, 6- hexanediol (Figures 4F, G). Although the percentage of PrD6 animals that did not exhibit condensates at 3% of 1, 6- hexanediol was only 20% less than PrD6 animals in heat shock, the condensates that resisted the alcohol in PrD6 gonads appeared smaller and scattered when compared to condensates in PrD6 heat-shocked animals. The TIAR-1 condensates in the oocytes of wild-type and PrD6-mutant animals did not dissolve with 1, 6- hexanediol (data not shown). These data demonstrated that the TIAR’s PrD is important for forming strong TIAR-1 condensates.

### CGH-1 condensates assembled efficiently in the germline of PrD6-mutant animals

**FIGURE 5:**
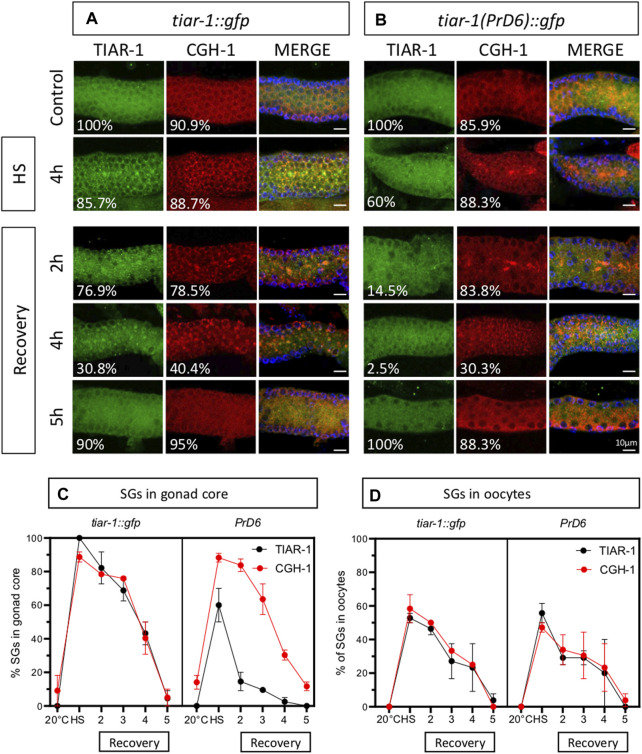
The prion-like domain of TIAR-1 does not influence the formation and disassembly of CGH-1 condensates. *tiar-1::gfp* and PrD6-mutant one-day-old animals were grown in normal conditions (control) or exposed to heat shock (4 h at 31°C). Some animals were kept at 20°C after the stress to assess their recovery. After stress or recovery period, gonads were dissected, fixed and coimmunostained with anti-GFP (green), anti-CGH-1 (red), and DAPI (cyan, shown in merge images) to visualize granules and DNA, respectively. **(A, B)** Confocal microscopy images were taken of gonads from heat-shocked and recovered animals at indicated times. The percentage of animals displaying the phenotype is indicated in each image. The bar indicates 10 μm and is the same scale for all images. **(C, D)** Graphs showing the percentage of animals forming condensates in the gonad core **(C)** or oocytes **(D)**, respectively at indicated conditions and time points. At least two independent experiments were performed, each involving a minimum of 25 animals per condition. The Bonferroni multiple comparison test was employed for statistical after conducting a 2-way ANOVA.

To determine whether the association of other stress granules components was also affected by the TIAR-1’s PrD domain mutation, we use CGH-1 as an additional SGs marker (Boag et al., 2005). Previously, we showed that TIAR-1::GFP co-localized with CGH-1 in condensates that exhibited stress granules features (Huelgas-Morales et al., 2016). We subjected *tiar-1::gfp* and the PrD6-mutant one-day-old animals to control conditions or 4 h of heat shock. After heat shock, some animals were processed immediately while others were set in place at 20°C to test their recovery. After treatment or recovery time, animals’ gonads were dissected, fixed and stained with CGH-1 and GFP antibodies. As we described in previous figures, gonad core TIAR-1::GFP condensates assembled efficiently after 4 h of heat shock and disassembled gradually for 5 h while condensates in PrD6 mutant animals did not assemble efficiently and most of them disassembled quickly after 2h of heat shock (85.5%) (Figures 5A–C). On the other hand, CGH-1 gonad core condensates assembled efficiently after heat shock in both *tiar-1::gfp* and PrD6-mutant animals (Figures 5A–C) and disassembled gradually during the following 5 h after heat shock. We did not observe any differences in assembling and disassembling efficiency in oocytes’ condensates for any of the strains or SGs markers (Figure 5D). These data suggest that the mutation in the PrD of TIAR-1 affects its association with SGs but does not affect the association of other components with these condensates.

**FIGURE 6:**
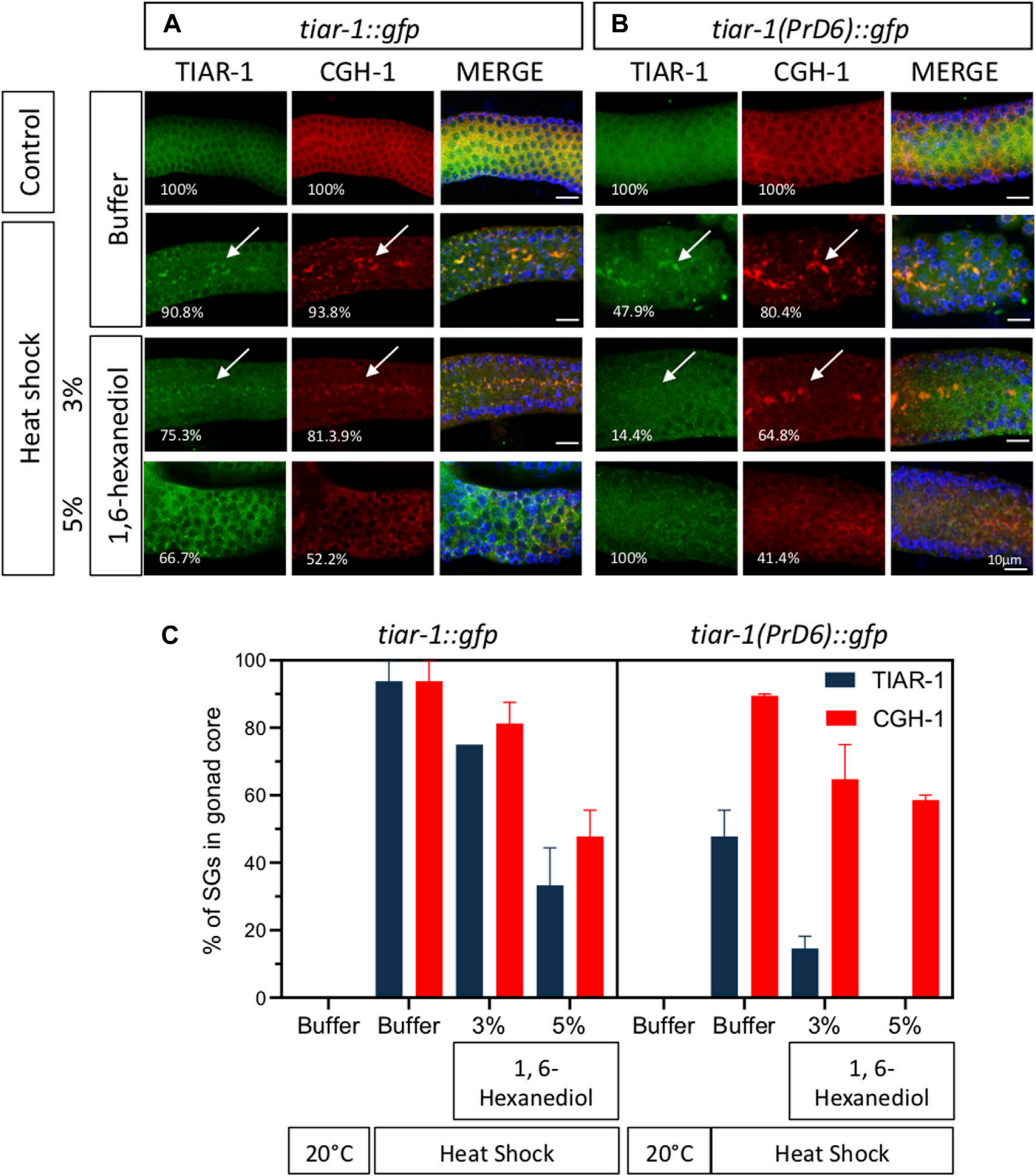
Most of the CGH-1 condensates in PrD6-mutant animals are still present after treatment with 1,6-hexanediol. *tiar-1::gfp* and PrD6-mutant one-day-old animals were grown in normal conditions (control) or exposed to heat shock (4 h at 31°C). After treatment, gonads were extruded in only buffer, or buffer containing 3% or 5% 1,6-hexanediol, subsequently, samples were coimmunostained with anti-GFP (green), anti-CGH-1 (red), and DAPI (cyan, shown in merge images) to visualize granules and DNA, respectively. **(A, B)** Confocal microscopy images were captured of the indicated conditions. The percentage of animals displaying the phenotype is indicated in each image. Condensates are indicated by white arrows. The bar indicates 10 μm and is the same scale for all images. **(C)** Graph illustrating the percentage of animals exhibiting TIAR-1 and CGH-1 condensates in the gonad core under each condition. At least two independent experiments were performed, each involving a minimum of 30 animals per condition. The Bonferroni multiple comparison test was employed for statistical after conducting a 2-way ANOVA.

To test the strength of the association of CGH-1 with SGs, we exposed *tiar-1::gfp* and PrD6-mutant animals to control and heat shock conditions (4 h) and dissected their gonads in the presence of 3 or 5% of 1,6-hexanediol followed by fixation and immunostaining with anti-GFP and anti-CGH-1. Most of the TIAR-1::GFP and CGH-1 condensates were resistant to 3% of 1,6-hexanediol, but approximately half of them disassembled at a concentration of 5% (Figures 6A, C). In PrD-6 mutant animals most of the TIAR-1 condensates disassembled in the presence of 3% or 5% of 1, 6-hexanediol meanwhile CGH-1 condensates were still observed under these conditions although in a lower proportion (Figures 6B, C). Our data suggest that the TIAR-1 PrD mutation associates with SGs with less strength than other components.

### PrD6 TIAR-1 mutant animals showed embryonic lethality, lower fertility and a shorter lifespan than wild-type animals

**FIGURE 7:**
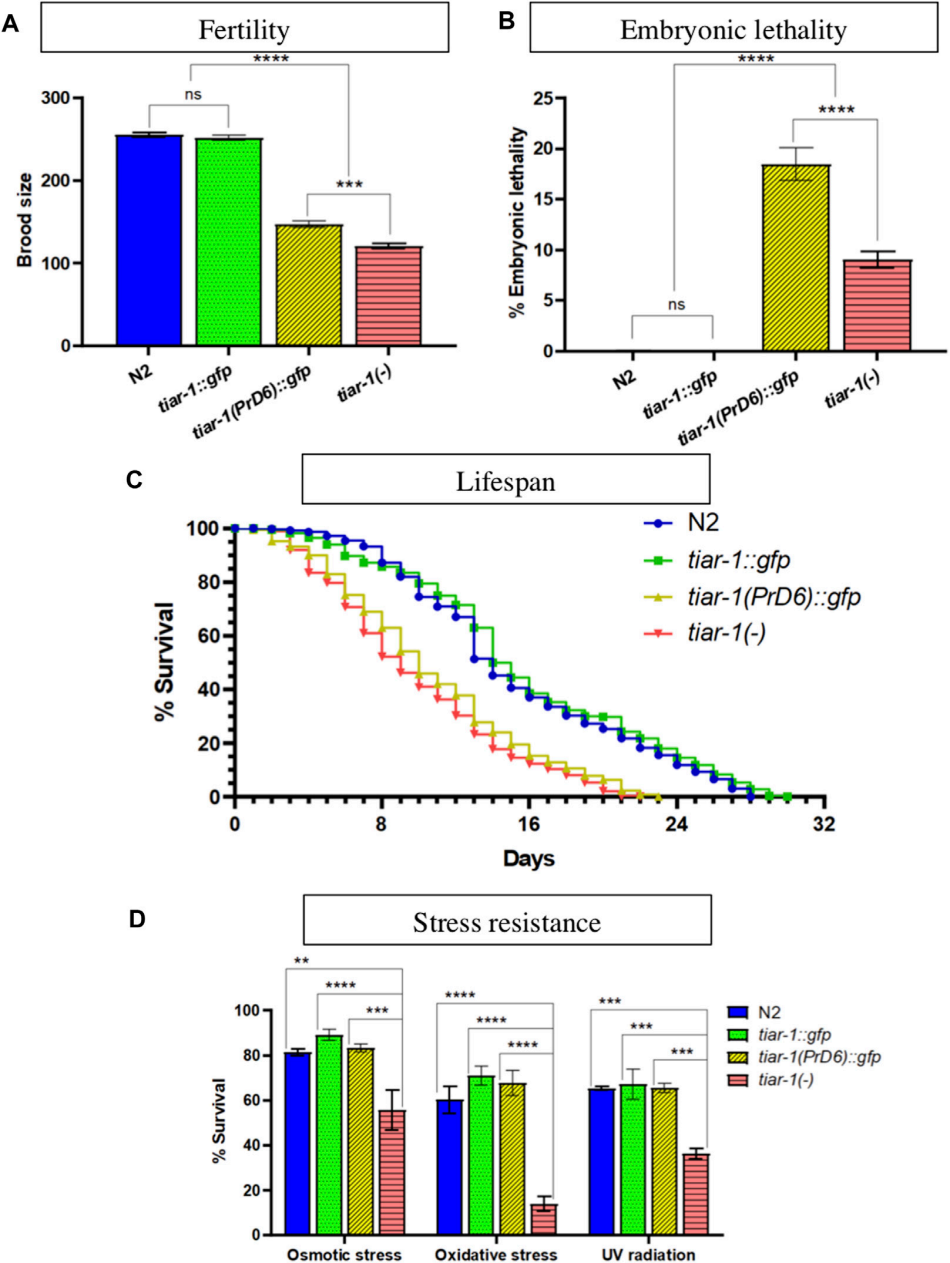
The TIAR-1 PrD is not essential for stress resistance. **(A, B)** Graphs showing the brood size and embryonic lethality of the N2 (wild-type), *tiar-1::gfp*, *tiar-1(PrD6)::gfp* and knock-out *tiar-1(tn1543)*. Individual hermaphrodites were selected at the mid-L4 stage and transferred to new plates every 24 h over the course of 3 days. The plates were assessed for the presence of dead embryos and the number of surviving offspring was scored. Embryos not hatching within 24 h after being laid were considered dead. For each genotype two independent experiments are shown. **(C)** A graph illustrating the percentage of living animals over time. Hermaphrodites at the mid-L4 stage of the indicated genotypes were placed in agar plates with bacteria. The animals were transferred to fresh plates every 24 h for a period of 4 days and maintained at a temperature of 20°C throughout the assay. Once the animals stopped producing offspring, they were kept on the same plate for the duration of the experiment. Death was defined as lack of any wild-type visible movement, lack of movement after touching the tail and head with a platinum wire and the loss of pharyngeal pumping, the animals that showed these parameters were considered dead. At least two independent experiments were conducted with 700 animals in total for each indicated genotype. **(D)** Graph showing the percentage of alive animals of the indicated genotypes after exposing them to the indicated stresses. For details see materials and methods. For each condition four independent experiments are shown. The Tukey multiple comparison test was employed for statistical after conducting a 2-way ANOVA and the results are indicated as follow: ****p < 0.0001, ***p < 0.001, **p < 0.01.

We assessed the impact of the mutation in TIAR-1′s PrD on the phenotypes associated with the absence of its gene. We and others have reported a small brood size and high embryonic lethality when studying the *tiar-1* knockout alleles (Silva-García and Estela Navarro, 2013; Rousakis et al., 2014; Huelgas-Morales et al., 2016). We compared brood size and embryonic lethality between the wild-type, *tiar-1::gfp* transgene, the *tiar-1(tn1543)* knock-out (KO) allele and the *tiar-1(syb4286)* PrD6 mutant *allele*. In fertility assays, both *tiar-1*-mutant alleles exhibited a decrease in brood size when compared to wild-type strains (N2) and *tiar-1::gfp* (Figure 7A). We observed that *tiar-1(tn1543)* KO-mutant animals had fewer progeny than the PrD6-mutant animals; although the difference was significant, it was also very small (Figure 7A). Wild-type and *tiar-1::gfp* animals displayed minimal embryonic lethality, whereas the *tiar-1(tn1543)* KO strain exhibited a small percentage of embryonic lethality (9%); unexpectedly, we observed an increase in embryonic lethality in the PrD6-mutant animals (18.5%) (Figure 7B).

It was previously published that the lifespan of *tiar-1(RNAi)* animals is shorter than that of wild-type (Rousakis et al., 2014), therefore we tested PrD6-mutant animals’ life span. Synchronized wild-type and *tiar-1*-mutant alleles were selected as L4 larvae and transferred to new plates each day for the following 4 days to isolate them from their progeny. The animals were observed every day to quantify the animals that were alive. We noted that the *tiar-1::gfp* strain exhibited a lifespan similar to that of the wild-type strain, while both *tiar-1*-KO and PrD6-mutant animals displayed shorter lifespans, which were comparable to each other (Figure 7C). Our data provides evidence that the prion-like domain of TIAR-1 may play a significant role in fertility, embryonic lethality, and lifespan.

Previous reports indicate that *tiar-1* protects *C. elegans* from stress (Rousakis et al., 2014). Therefore, we evaluated the resistance of PrD6-mutant animals to osmotic and oxidative stress, as well as to UV radiation. We subjected one-day-old wild-type, *tiar-1::gfp* transgene, *tiar-1(tn1543)* and *tiar-1(syb4286)* PrD6 mutant animals to osmotic (400 mM NaCl for 24 h) or oxidative (5 mM NaAsO_2_ for 48 h) conditions. For UV radiation exposure (0.2 J/cm^2^), 5-day-old animals were employed (Rousakis et al., 2014). Above 80% of wild-type and *tiar-1::gfp* animals were resistant to osmotic stress while more than 60% of them resisted exposure to oxidative stress and UV radiation (Figure 7D). In contrast, the *tiar-1* knockout animals were partially sensitive to exposure to osmotic stress (55%) and UV radiation (36%) exposure. In particular, *tiar-1(tn1543)* KO animals were very sensitive to oxidative stress, and only 14% of them survived this condition (Figure 7D). We observed that the resistance of PrD6-mutant animals to stress was comparable to that of wild-type and *tiar-1::gfp* transgenic animals (Figure 7D). Our data suggest that the prion domain region of TIAR-1 may not play a significant role in animals’ stress protection. It is also possible that TIAR’s PrD6 protein’s weak association with SGs is sufficient to fulfill TIAR-1′s functions under stressful conditions.

### The TIAR-1 prion domain is not required to trigger germ cell apoptosis under stressful conditions

**FIGURE 8:**
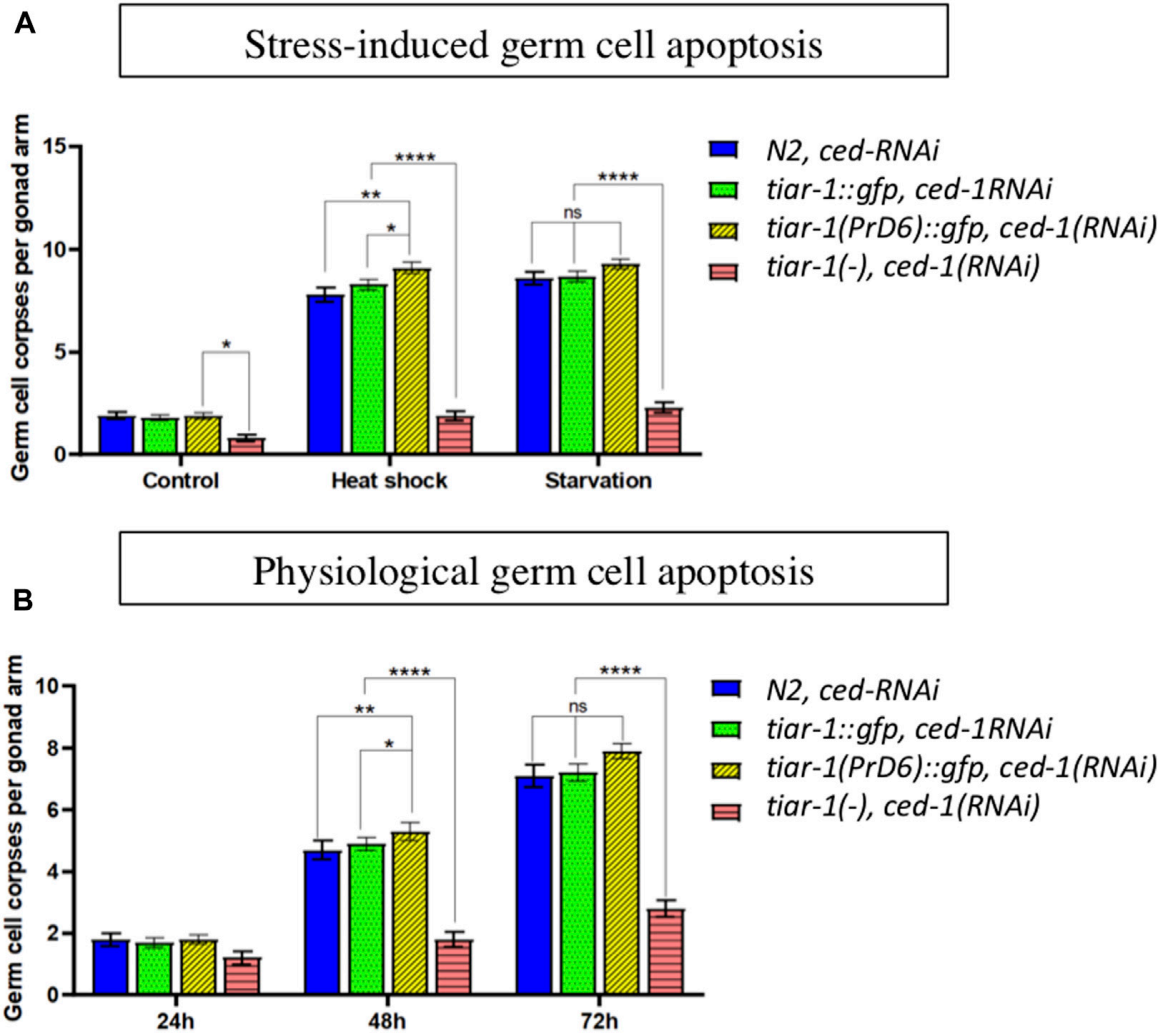
The TIAR-1 PrD is not essential for stress-induced germ cell apoptosis. **(A)** A graph showing the number of germ cell corpses when one-day-old animals of the indicated genotypes were exposed to different types of stress. Previously, mid-L4 stage hermaphrodites were exposed to dsRNA of *ced-1* gene for 24 h to facilitate germ cell corpses observation. After treatments, animals were observed under Nomarski microscopy to quantify germ cell corpses. **(B)** A graph showing germ cell corpse number as animals age. Animals of the indicted phenotypes were kept under control conditions, and each day were observed under Nomarski microscopy to quantity germ cell corpses. One-day-old animals are indicated as 24 h. For each genotype three independent experiments are shown, with the exception of *tiar-1* KO, in which only one experiment was performed. Tukey multiple comparison test, after 2 way ANOVA, were employed and the results are indicated as follow: ****p < 0.0001, **p < 0.01, *p < 0.05.

Previously, our laboratory reported that TIAR-1 is necessary to induce germ cell apoptosis under stress conditions (Silva-García and Estela Navarro, 2013). We tested stress-induced germ cell apoptosis in the PrD6-mutant animals and compared it with wild-type and the knockout (KO) strain. To facilitate the detection of germ cell corpses, we performed RNAi in the *ced-1* gene, which is required for the degradation of the germ cell corpse (Zhou et al., 2001). We subjected the animals of the different strains and alleles to control conditions (20°C and bacteria), to heat shock (3 h at 31°C with 1 h at 20°C for recovery time) or starvation (no bacteria for 6 h). The wild-type animals showed stress-induced germ cell apoptosis after heat shock or starvation, as we previously reported (Salinas et al., 2006) (Figure 8A). The *tiar-1(tn1543)* KO animals were unable to induce germ cell apoptosis under heat shock or starvation conditions, as we previously published (Figure 8A) (Silva-García and Estela Navarro, 2013). In contrast, we observed that the PrD6-mutant animals showed an increase in germ cell apoptosis under heat shock and starvation conditions similar to the wild-type and *tiar-1::gfp* transgene animals (Figure 8A). These data suggest that the prion-like domain is dispensable for TIAR-1 function during apoptosis.

Physiological germ cell apoptosis typically occurs during *C. elegans* oogenesis*,* and as the animals age, this occurrence increases significantly (Gumienny et al., 1999). We tested the levels of physiological germ cell apoptosis in null and PrD6 *tiar-1* adult animals for 3 days and compared it with that of wild-type animals. We observed that the *tiar-1(tn1543)* KO mutant animals exhibited reduced levels of physiological apoptosis, which became more pronounced as the animals aged (Figure 8B). In contrast, the PrD6-mutant animals displayed an increased physiological apoptosis that paralleled the aging process, similar to the patterns observed in wild-type and *tiar-1::gfp* transgenic animals (Figure 8B). Our results suggest that the PrD region is not important for TIAR-1 function during apoptosis.

## Discussion

The TIA1 family of proteins has been extensively studied for their role in promoting stress granules condensation, as well as their involvement in other cellular processes, such as RNA splicing (Kedersha et al., 1999; Del Gatto-Konczak et al., 2000; Damgaard and Lykke-Andersen, 2011), mRNA translation control (Piecyk et al., 2000), viral and bacterial infection (Albornoz et al., 2014), control of cell proliferation (Reyes et al., 2009), as tumor suppressors (Izquierdo et al., 2011), and in diseases such as Amyotrophic Lateral Sclerosis (ALS) (Mackenzie et al., 2017) and Welander Distal Myopathy (WDM) (Hackman et al., 2013; Klar et al., 2013). However, the connection between the roles of TIA1 proteins in condensation and their other cellular functions has received relatively little study. To explore this, we disrupted the structure of two hypothetical α-helices within the prion-like domain of the *C. elegans* TIAR-1 protein to influence its condensation properties and studied the effects of this modification on the nematode’s phenotype. We observed that the mutated version of TIAR-1 was still able to form condensates; however, they did not exhibit the properties of the wild-type granules. The condensates formed by the mutated TIAR-1 protein exhibited lower assembly than the wild-type protein. Furthermore, when TIAR-1 PrD6 condensates did form, they were not well assembled or organized. After stress and on exposure to 1, 6-hexanediol, condensates from the PrD6-mutated version of TIAR-1 dissociated faster than those of wild-type version of the protein, demonstrating that the two hypothetical α-helices within the prion-like domain of TIAR-1 are important for its condensation under stress conditions in the *C. elegans* germline. We observed that PrD6-mutant animals, similar to the TIAR-1 KO nematodes, continued showing fertility problems, a lower lifespan, and increased embryonic lethality. Unexpectedly, inefficient TIAR-1 condensation did not affect the animals’ protection from stress, or stress-induced germ cell apoptosis.

### The prion-like domain of TIAR-1 has two hypothetical α-helices that play crucial roles in its localization and stability within condensates

Through the sequence analysis of the prion-like domain of *C. elegans* TIAR-1, we found that, within its carboxy-terminal, there are 29 residues enriched in polar (Q, N) and aromatic residues (Y, W) that form two hypothetical α-helices that are conserved among TIAR-1 homologs (Figure 1). Structured regions in prion-like domains involved in liquid-liquid phase separation (LLPS) are often reported to be amyloid-like sheets or LARKs (Mittag and Parker, 2018; Gomes and Shorter, 2019), while α-helices are less common. However, conserved helicoidal structures that promote condensation have recently been reported in human proteins TDP-43 and in Musashi-1 and -2 studies (Conicella et al., 2016; Conicella et al., 2020; Chiu et al., 2022).

We disrupted the α-helices of the PrD of TIAR-1 protein by introducing interspersed substitutions of six residues (S, Q, Y, Q, S, and Q) with prolines (Figure 1). We observed that the mutant TIAR-1 protein exhibited less efficient condensation during stress compared to the wild-type version (Figure 2). We defined two phenotypes of TIAR-1 condensates in the gonad core, which we think represented different levels of assembly: defined SGs and dispersed SGs. We considered the defined SGs as the stronger, end-point phenotype in which the majority of TIAR-1::GFP foci are observed along the midline of the gonad core. We considered that dispersed SGs correspond to a transitional stage into defined SGs, in which we observed smaller and fewer foci, which can be found at the midline and/or other areas of the rachis (Figure 2).

We did not observe any differences in the distribution and assembly efficiency of TIAR-1 condensates when we extended exposure to heat shock in the gonad core of the PrD6-mutant animals increased. In contrast, there is an increased efficiency of TIAR-1 condensates assembly at the gonad core in extended starvation, which implies other mechanisms involved in SG assembly may be acting on these conditions. Additionally, we observed that the condensates formed during heat shock by the PrD6-mutated version of TIAR-1 disassembled at a faster rate than the granules formed by the wild-type TIAR-1 protein after exposure to stress (Figures 3, 4).

These findings suggest that the integrity and proper functioning of the α-helices in the PrD region are essential for the regulation of TIAR-1 condensation dynamics and its subsequent removal from these condensed structures. SGs clearance compromises a critical step, because it allows the cells to return to their regular functions. Conversely, the inability to maintain SGs condensates when the cells are still in the process of returning to their basal stage after stress can result in the activation of cell functions that may alter cellular performance.

Unexpectedly, we observed that CGH-1, a SGs marker, formed condensates with the same efficiency during heat shock in both wild-type and PrD6-mutant animals (Figure 5). In contrast to TIAR-1 PrD6 mutant condensates, CGH-1 condensates dissociated slowly from SGs and are as resistant to 1, 6-hexanediol as the wild type condensates (Figures 5, 6). Our data suggest that only TIAR-1 is affected by the PrD6 mutation, while other SGs components remain unaffected by this change. However, it is advisable to use other SGs markers to assess whether condensates in PrD6-mutant animals remain unaffected.

We previously reported that TIAR-1 is not necessary for SGs formation in the oocytes (Huelgas-Morales et al., 2016), and consequently we observed that PrD6 TIAR-1-mutant animals still form condensates that did not disassemble with 1, 6-hexanediol. Oocyte-specific proteins could promote the observed TIAR-1 and PrD6-mutant TIAR-1 association to SG’s and resistance to disassembly. Large condensates formed in arrested oocytes of *C. elegans* gonads depleted of sperm or when animals are exposed to different types of stress (Elaswad et al., 2022). Proteins found associated with these large oocyte condensates can show different liquid phases. The intrinsically disordered protein MEG-3 shows gel-like properties in oocytes (Elaswad et al., 2022), similarly to the properties than show on P granules during embryogenesis, in which play a key role in maintaining condensates together during cell divisions (Putnam et al., 2019). The RNA binding protein MEX-3 shows several gel-like properties but is more dynamic than MEG-3. PUF-5, a translational repressor, promotes the condensation of MEX-3 and MEG-3 into large RNP granules. These proteins may contribute to the resistance of TIAR-1 SGs in oocytes. Nevertheless, there is much to discover about the liquid condensation phases observed in the *C. elegans* germline.

### Prolines play a key role in the condensation of the TIA1 protein family

Prolines play a key role for LLPS in human TIA1 (Ding et al., 2021). The low complexity domain of TIA1 contains multiple prolines that are mutated in patients with diseases such as Amyotrophic Lateral Sclerosis (ALS) and Fronto-Temporal Dementia (FTD). Specifically, the substitution of proline for leucine (P352L and P362L) in TIA1 has been found to promote the aggregation and formation of amyloid fibrils in the mutated protein. Contrariwise, the substitution of 11 proline residues in the LCD of TIA1 for alanine resulted in aggregation and delayed disassembled SGs (Ding et al., 2021). Based on their findings, Ding et al., proposed that proline residues play a crucial role in inhibiting the aggregation of the prion-like domain in TIA1. This could explain why the introduction of prolines to the PrD region of TIAR-1 in *C. elegans* made TIAR-1-mutated condensates more liquid.

Another α-helix structure that participates in modulating LLPS is found in the RNA-binding protein TDP-43. This RNA binding protein forms cytoplasmic neuronal inclusion through its C-terminal domain in the Amyotrophic Lateral Sclerosis Disease (ALS). It was reported that the C-terminal domain of TDP-43 folds into a partial α-helix structure. When a single proline substitution (A316P) was introduced into this region to interfere with the structure, the α-helix structure was disrupted, and the modified protein was unable to form aggregates (Conicella et al., 2016).

To disrupt the hypothetical α-helices of the PrD of the TIAR-1 protein, we exchanged two serines and three glutamines for prolines. Although the prediction is that the introduction of proline residues would completely disrupt the α-helix structure, the lack of these serine or glutamine residues could also have a consequence in the properties of TIAR-1 mutant protein. Glutamine and serine residues contribute slightly to reducing fluidity in condensates of the FUS protein, another RNA-binding protein that associates with SGs (Wang et al., 2018). However, although serine and glutamine residues mutation in the FUS protein disrupted cross-beta sheet interactions, these did not exert a strong influence on phase separation *in vitro*.

Another residue that is frequently found in prion-like domains is tyrosine (Wang et al., 2018). The specific mechanisms governing phase separation can vary depending on the context and the proteins involved; however it has been observed that it could be governed by multivalent interactions among tyrosine residues from prion-like domains and arginine residues from RNA-binding domains. Here we exchanged one tyrosine residue for proline, and it is possible that this substitution might have affected the interaction of the prion-like domain of TIAR-1 with its RNA binding domain. Tyrosines are considered important for the condensation of FUS and other LCD-containing proteins (Wang et al., 2018). In *C. elegans*, the substitution of tyrosines with glycines in the prion-like domain of TIAR-2 completely abolished the association of this protein with SGs (Andrusiak et al., 2019).

Indeed, the PrD6 TIAR-1-mutant protein retains certain domains that could potentially contribute to its interaction with other components of the SGs and facilitate its condensation. Furthermore, it is conceivable that residues within the prion-like domain, other than the mutated region, may also play a significant role in TIAR-1 condensation. It was shown in a study by Lin et al. (2015) that RNA Recognition Motifs (RRMs) domains also play a role in phase separation. Additionally, it was observed that the presence of RRM lowers the saturation concentration of proteins required for condensation (Molliex et al., 2015). The role of TIAR-1′s RRMs in condensation may also explain why the CGH-1 protein forms condensates in stressed *tiar-1* PrD mutant animals but cannot form them in *tiar-1* knock-out animals (Rousakis et al., 2014). This suggests that the RRM domains of TIAR-1 might facilitate the relocalization of helicase CGH-1 to SGs, either through direct contact or by facilitating the RNA docking of the latter.

The PrD6 *tiar-1*-mutant and the *tiar-1*-KO animals exhibited similar phenotypes concerning fertility and lifespan, nonetheless displaying notable differences in stress-related phenotypes.

Similar to *tiar-1* KO animals, PrD6-mutant animals demonstrated an affected fertility and a lower lifespan but they unexpectedly had a higher embryonic lethality (Figures 7A, B) (Rousakis et al., 2014; Huelgas-Morales et al., 2016; Andrusiak et al., 2019). It is surprising that the PrD6-mutant animals show a more significant impact on embryonic lethality than the knockout. It is possible that TIAR-1 plays an important role in the assembly of condensates, such as the P granules, during embryogenesis. However, it is also possible that the TIAR-1 PrD6-fusion protein has certain folding characteristics that can influence the role of TIAR-1 in embryogenesis. In mammals, the loss-of-function TIA-1 and TIAR affects embryogenesis and germ cell development (Beck et al., 1998; Piecyk et al., 2000), while TIAR overexpression disturbs embryonic development (Kharraz et al., 2010). While fertility and embryonic development naturally progress in the absence of stress, our studies suggest that the ability of the TIA1 family of proteins to form condensates may be associated with these processes. Although it is also plausible that fertility and embryonic development do not require TIAR-1 condensation and the role of the PrD domain in these processes remains to be discovered. Further exploration of the role of TIAR-1 condensation in embryogenesis, fertility and lifespan promises to be an intriguing avenue of research.

Unexpectedly, *tiar-1* KO phenotypes related to stress survival and apoptosis (Rousakis et al., 2014; Huelgas-Morales et al., 2016; Andrusiak et al., 2019) were recovered in PrD6-mutant animals (Figures 7D, 8) suggesting that the association of TIAR-1 with condensates is not essential for protecting the organism from stress or stress-induced apoptosis. It is indeed plausible that the cellular functions affected by the TIAR-1 prion-like mutation do not necessarily require that all TIAR-1 proteins be condensed and recruited into condensates and that small amounts of protein are sufficient for the protein to regulate its targets. On the other hand, the other SGs components may be sufficient to protect the organism from stress.

The precise size and composition of a condensate required to perform its function remains an area of ongoing research and is currently not fully understood. Our understanding of biophysical properties and their impact on physiological outcomes is expanding as we discover the association between numerous cellular processes and liquid-like states. This is only the beginning of our appreciation for these connections and how they can be modified under pathological conditions. Further research is needed to elucidate the intricate connections and potential interplay between the condensation properties of the TIA1 family of proteins and their broader cellular functions.

## Data Availability

The raw data supporting the conclusion of this article will be made available by the authors, without undue reservation.

## Supplementary material

SUPPLEMENTARY FIGURE S1 TIAR-1 condensates are disrupted in the presence of 5% 1. 6-hexanediol. **(A-C)** Pictures of selected areas of the gonad core were taken. One-day-old *tiar-1::gfp* and *PrD6*-mutant hermaphrodites were exposed to heat shock (4 hrs at 31°C, B and C) or kept at 20°C (control, A). After treatment, gonads were extruded on buffer (A and B) or in presence of 5% 1,6-hexanediol (C). Samples were covered with a coverslip and observed immediately under the epifluorescence microscope to score for condensates. The percentage of animals showing condensates is shown in each picture. At least two independent experiments representing at least 50 animals for each condition.
