# Supplementary figures and images for: Two predicted α-helices within the prion-like domain of TIAR-1 play a crucial role in its association with stress granules in Caenorhabditis elegans
Source: Front Cell Dev Biol. 2023 Dec 15;11:1265104. doi: 10.3389/fcell.2023.1265104 (PMC10757852; doi:10.3389/fcell.2023.1265104)

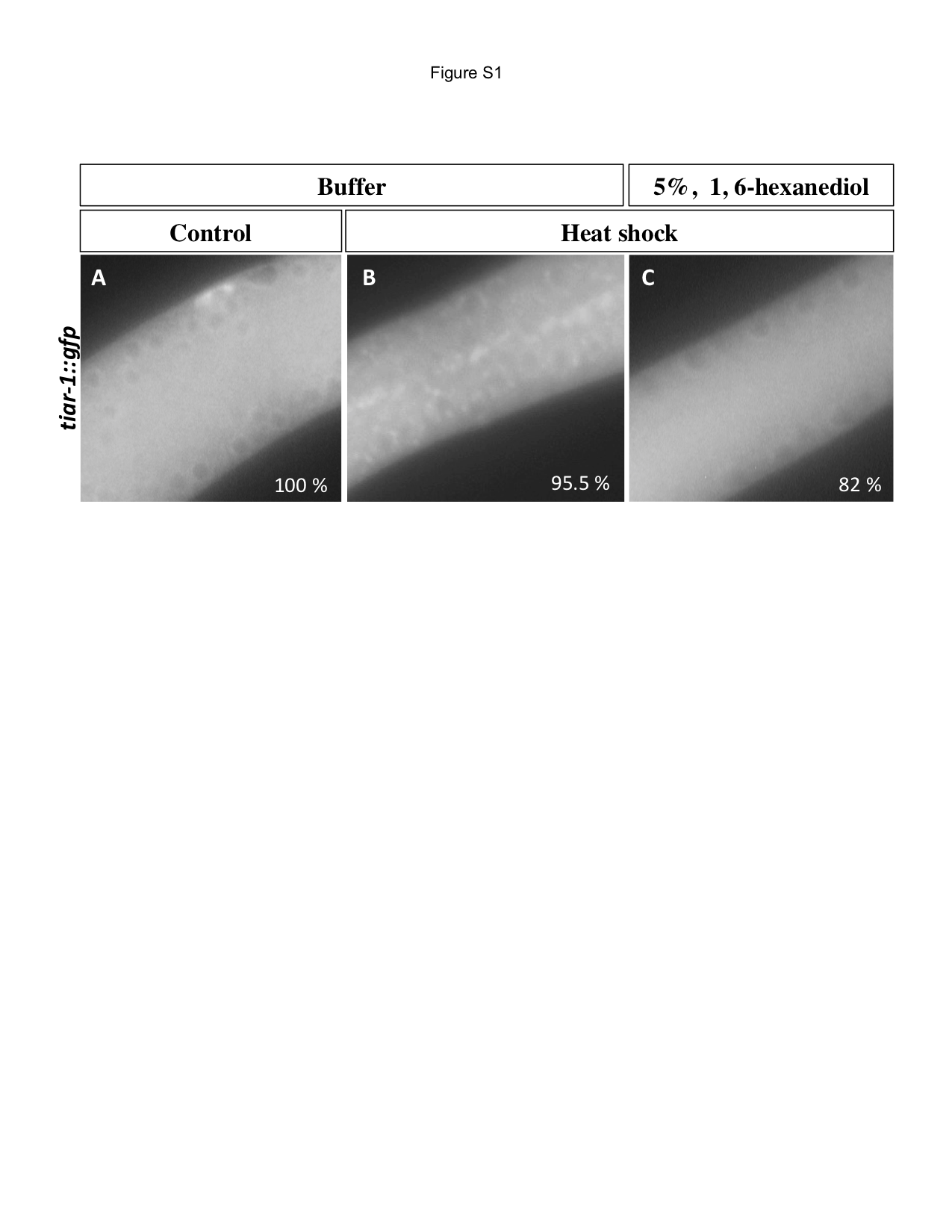

Supplement: Supplementary file 1 [file Image1.TIFF]
